# MEG3-Mediated Oral Squamous-Cell-Carcinoma-Derived Exosomal miR-421 Activates Angiogenesis by Targeting HS2ST1 in Vascular Endothelial Cells

**DOI:** 10.3390/ijms25147576

**Published:** 2024-07-10

**Authors:** Chia-Yun Huang, Sung-Tau Chou, Yuan-Ming Hsu, Wan-Ju Chao, Guan-Hsun Wu, Jenn-Ren Hsiao, Horng-Dar Wang, Shine-Gwo Shiah

**Affiliations:** 1 National Institute of Cancer Research, National Health Research Institutes, Miaoli 350401, Taiwan; s700701166@gmail.com (C.-Y.H.); sungtau@gmail.com (S.-T.C.); ymhsu@nhri.edu.tw (Y.-M.H.); wanju0729@nhri.edu.tw (W.-J.C.); dreannblue0213@nhri.edu.tw (G.-H.W.); 2 Institute of Biotechnology, National Tsing Hua University, Hsinchu 300044, Taiwan; hdwang@life.nthu.edu.tw; 3 Head and Neck Collaborative Oncology Group, Department of Otolaryngology, National Cheng Kung University Hospital, College of Medicine, National Cheng Kung University, Tainan 704302, Taiwan; hsiaojr@ncku.edu.tw; 4 Program in Environmental and Occupational Medicine, Kaohsiung Medical University, Kaohsiung 80708, Taiwan; 5 Cancer Center, Wan Fang Hospital, Taipei Medical University, Taipei 116079, Taiwan

**Keywords:** microRNA, long non-coding RNA (lncRNA), oral squamous cell carcinoma (OSCC), exosome, angiogenesis, miR-421, HS2ST1

## Abstract

Exosomal microRNAs (miRNAs) from cancer cells play a key role in mediating the oral squamous cell carcinoma (OSCC) microenvironment. The objective of this study was to investigate how the long non-coding RNA (lncRNA) MEG3 affects OSCC angiogenesis through exosomal miR-421. Global miRNA microarray analysis and quantitative real-time PCR (qRT-PCR) were performed to determine the level of miRNAs in OSCC cell-derived exosomes. Cell migration, invasion, tube formation, immunohistochemistry, and hemoglobin concentrations were used to study the effects of exosomal miR-421 in angiogenesis. Western blotting was used to determine the expression level of HS2ST1 and VEGFR2-related downstream proteins. MiRNA array and qRT-PCR identified the upregulation of miR-421 in OSCC cell-derived exosomes. Furthermore, exosomal miR-421 can be taken up by human umbilical vein endothelial cells (HUVECs) and then target HS2ST1 through VEGF-mediated ERK and AKT phosphorylation, thereby promoting HUVEC migration, invasion, and tube formation. Additionally, forced expression of the lncRNA MEG3 in OSCC cells reduced exosomal miR-421 levels and then increased HS2ST1 expression, thereby reducing the VEGF/VEGFR2 pathway in HUVECs. Our results demonstrate a novel mechanism by which lncRNA MEG3 can act as a tumor suppressor and regulate endothelial angiogenesis through the exosomal miR-421/HS2ST1 axis, which provides a potential therapeutic strategy for OSCC angiogenesis.

## 1. Introduction

Oral squamous cell carcinoma (OSCC), with a 5-year survival rate of 50–60%, is the major cause of oral cancer morbidity and mortality and has become a major global health challenge [1,2]. In the past 20 years, despite therapeutic improvements, the prognosis of OSCC has not improved significantly, mainly due to late diagnosis, frequent locoregional recurrence, and metastasis [3]. Angiogenesis has been reported to be associated with tumor metastasis, disease progression, and poor prognosis in OSCC [4]. It involves the complex biological process of new blood vessel formation involving interactions between vascular cells and the extracellular environment [5]. Tumor cells can induce angiogenesis by secreting various growth factors, cytokines, proteases, and even exosomes to the surrounding environment, providing oxygen and nutrients through new blood vessels to maintain tumor growth [5]. However, the detailed mechanisms between tumorigenicity and angiogenesis in OSCC are not fully understood.

Heparan sulfate proteoglycans (HSPGs) are a group of distinct glycoproteins, found on the cell surface and in the extracellular matrix (ECM), composed of a core protein with one or more covalently linked heparan sulfates (HSs), a type of glycosaminoglycan (GAG) [6,7]. Both HS GAGs and HSPGs bind various ligands and play important roles in normal physiology and pathological effects, including embryonic development, homeostasis, inflammatory responses, microbial infection, angiogenesis, and tumor initiation and progression [7,8]. However, HS chains appear to determine the binding affinity to various ligands, such as growth factors, cytokines, chemokines, and ECM components [7,9]. HSs are unbranched polysaccharides composed of uronic acid (glucuronic acid, GlcA, or iduronic acid, IdoA) and N-acetyl-glucosamine (GlcNAc) disaccharide repeats with various sulfation modifications [10,11]. The sulfation of HS is an important post-translational modification catalyzed by 2-O, 3-O, and 6-O sulfotransferases. 2-O-sulfation within HS is catalyzed by HS 2-O sulfotransferase (HS2ST1) and is critical for the participation of HS in various ligand-binding capabilities and signaling transductions [12,13]. Abnormal expression and function of HS2ST1 have been detected in breast cancer, where the upregulation of HS2ST1 is associated with reduced invasive behavior and the cancer stem cell phenotype [14,15]. Furthermore, it has been shown that the upregulation of HS2ST1 leads to conformational changes in HS and reduces basic fibroblast growth factor binding, resulting in reduced p44/42 MAPK and p38 MAPK activity, which correlates with a reduced invasive phenotype [14]. HSPGs are also present on the surface of vascular endothelial cells and contribute to the regulation of vascular permeability, immune cell trafficking, ligand binding, and blood vessel formation [16,17]. Although HS2ST1 plays a key role in cancer progression, its regulation and mechanisms involved in vascular endothelial cells have not been clearly elucidated.

It is widely recognized that tumor-derived exosomes possess the capacity to regulate cell–cell communication between the tumor and surrounding tumor microenvironment (TME) by transferring cargoes such as proteins, nucleic acids, and lipids [18]. One of the most important cargoes in exosomes is microRNAs (miRNAs), which belong to the family of single-stranded non-coding RNAs, are around 18–25 nucleotides long, and are involved in gene regulation at the post-transcriptional level [19]. Accumulating evidence suggests that tumor-derived exosomal miRNAs play a key role in stimulating endothelial cell migration and blood vessel formation that, in turn, support tumor progression and metastasis [20,21,22]. In this study, we found that miR-421 is abundantly present in OSCC-derived exosomes and is able to significantly induce tube formation in endothelial cells. MiR-421 has been shown to exert oncogenic and inhibitory effects on programmed cell death protein 4 in OSCC cells [23]. However, there are currently no studies in the literature on the related role of OSCC-derived exosomal miR-421 in human endothelial cells.

Here, we focused on exosomal miR-421 to study its effect on OSCC angiogenesis. We demonstrated that miR-421 could be transferred to human umbilical vein vascular endothelial cells (HUVECs) via exosomes secreted by OSCC cells. Furthermore, the internalized exosomal miR-421 targets HS2ST1 through the VEGF-mediated activation of ERK and AKT, thus promoting HUVEC migration, invasion, and tube formation. Maternally expressed gene 3 (MEG3), a long non-coding RNA (lncRNA), has been reported to function as a tumor suppressor gene in various solid cancers [24,25]. We further found that the downregulation of lncRNA MEG3 can lead to the upregulation of miR-421 in OSCC cells. In addition, treatment with exosomes derived from MEG3-overexpressing OSCC cells significantly increased the expression of HS2ST1 protein and, conversely, decreased the phosphorylation of VEGFR2, ERK, and AKT and the tube formation of HUVECs. Taken together, our results suggest that the lncRNA MEG3-mediated exosomal miR-421/HS2ST1 axis may be a new therapeutic target for OSCC angiogenesis.

## 2. Results

### 2.1. OSCC Cell-Derived Exosomal miR-421 Promotes HUVEC Tube Formation, Migration, and Invasion

**Figure 1:**
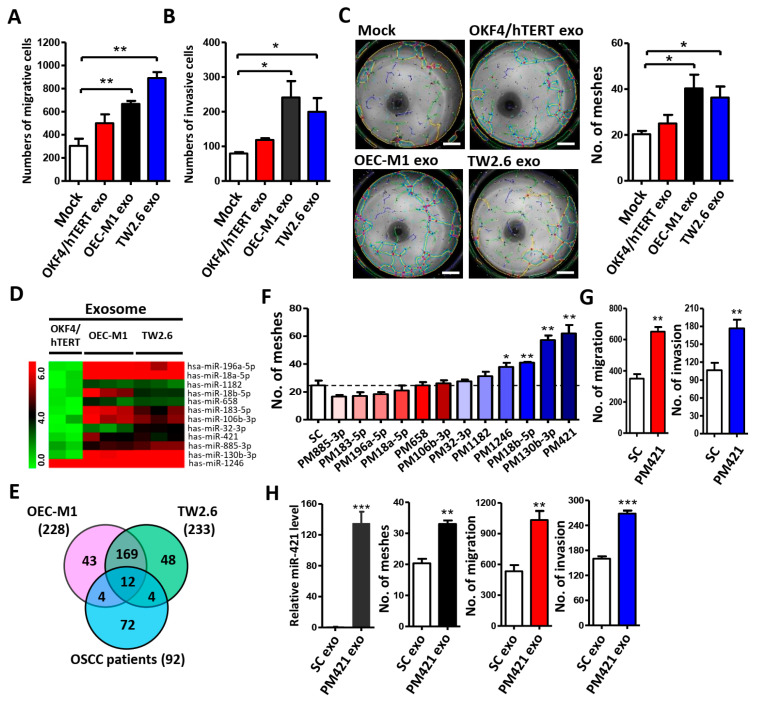
Exosomal miR-421 derived from OSCC cells promotes HUVEC migration, invasion, and tube formation. (**A**–**C**) Exosomes isolated from normal oral keratinocyte (OKF4/hTERT) or OSCC cells (OEC-M1 and TW2.6) were incubated with HUVECs for migration assay (**A**), invasion assay (**B**), and tube formation assay (**C**). Scale bar: 1 mm. (**D**) Heatmap of differentially expressed exosomal miRNAs. Two biological replicates (for OKF4/hTERT) and three biological replicates (for OEC-M1 and TW2.6) were used for Affymetrix miRNA array. The differentially expressed miRNAs were selected from OSCC-derived exosome (OEC-M1 and TW2.6) compared with normal oral keratinocytes (OKF4/hTERT). (**E**) Venn diagram of 12 differentially expressed miRNAs based on the intersection of OSCC-derived exosomal miRNAs with OSCC patient miRNA database (GSE37991). (**F**) Tube formation assay of HUVECs treated with 12 differentially expressed miRNA mimics. The dotted line represents the basal level of control (SC). (**G**) Transwell migration and invasion assay of HUVECs treated with miR-421 mimics (PM421). (**H**) Relative expression level of miR-421 in exosomes isolated from miR-421 (PM421) or scramble control (SC)-transfected OEC-M1 cells using quantitative-RT-PCR (*left*). Exosomes isolated from miR-421 (PM421) or scramble control (SC)-transfected OEC-M1 cells were incubated with HUVECs for migration, invasion, and tube formation assays (*middle* and *right*). Data are presented as mean ± SD; * *p* < 0.05; ** *p* < 0.01; *** *p* < 0.001.

To determine the function of exosome-mediated angiogenesis, we treated HUVECs with exosomes derived from OSCC cells (OEC-M1 and TW2.6) and transformed normal human keratinocytes (OKF4/hTERT) to study the effects of exosomes on their migration, invasion, and tube formation abilities. We used transmission electron microscope (TEM) (Supplementary Figure S1A), dynamic light scattering (DLS) (Supplementary Figure S1B), and Western blot analyses (Supplementary Figure S1C) to verify the presence of exosomes. These results indicate that the particles we isolated had typical sizes (50–120 nm), specific surface markers, and the characteristic morphology of exosomes. We then treated HUVECs with the obtained exosomes for 6 h. We found that exosomes derived from OSCC cells significantly increased HUVEC migration (Figure 1A), invasion (Figure 1B), and tube formation (Figure 1C) when compared with exosomes extracted from normal human keratinocytes (OKF4/hTERT), indicating that OSCC-derived exosomes have a remarkable ability to induce angiogenesis in endothelial cells.

Next, we used miRNA microarray analysis to explore the differentially expressed miRNAs in OSCC-derived exosomes compared with those in normal OKF4/hTERT cells. We found 228 miRNAs in OEC-M1 exosomes and 233 miRNAs in TW2.6 exosomes, as compared to normal OKF4/hTERT exosomes. Combining these two sets of results, we identified 181 miRNAs that were consistently differentially expressed in OSCC exosomes (Supplementary Table S1). In order to highlight the clinical significance of these miRNAs, we intersected these 181 miRNAs with OSCC patients whose miRNA expression data were deposited in the GEO database through our previous study (GSE37991) [26]. Finally, we screened 12 miRNAs that were upregulated in OSCC-derived exosomes with a filtered *p*-value ≤0.05 and >2-fold change (Figure 1D,E and Supplementary Table S2) compared with normal OKF4/hTERT exosomes. To determine whether these 12 selective miRNAs have direct effects on vascular endothelial cells, we directly transfected these 12 miRNA mimics (PMs) into HUVECs and evaluated their effects on tube formation ability. The results show that miR-421 and miR-130b-3p appear to exhibit a significant ability to induce tube formation of HUVECs (Figure 1F). Since exosome-derived miR-130b-3p-promoted angiogenesis has been reported in OSCC [27], miR-421 was chosen for subsequent experiments. We found that miR-421 not only stimulated tube formation but also significantly induced the migration and invasion of HUVECs in vitro (Figure 1G). Next, we transfected miR-421 mimic (PM421) into OEC-M1 to generate miR-421-enriched exosomes. As expected, HUVECs incubated with miR-421-enriched exosomes showed increased migration, invasion, and tube formation (Figure 1H), suggesting that miR-421 in OSCC cell-secreted exosomes plays a key role in promoting HUVEC angiogenesis.

### 2.2. miR-421 Induces Neovascularization In Vivo

**Figure 2:**
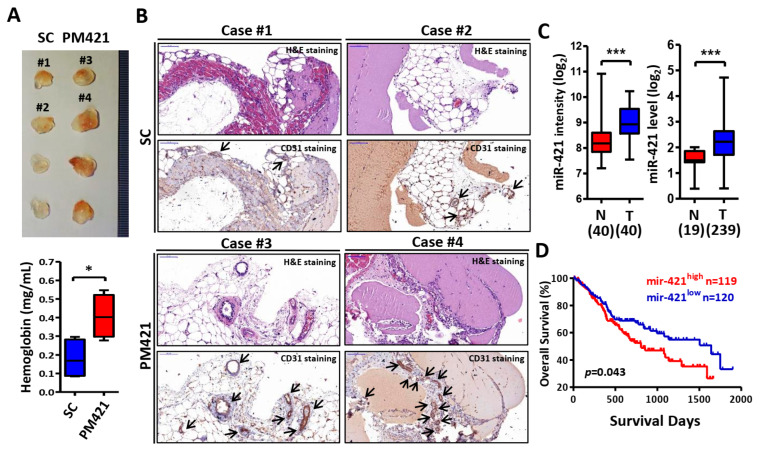
miR-421 induces neovascularization in vivo. (**A**) Image of Matrigel plugs from mice at 12 days after injection of Matrigel mixed with scramble control (SC) or miR-421 mimic (PM421) (*upper*). The hemoglobin concentration of plugs was determined and used as the angiogenesis index (*lower*). (**B**) Representative images of IHC staining for H&E and CD31 from Matrigel plugs. Selective case number is shown in (**A**) (scale bar: 100 μm). Arrowheads show the location of microvascular structures with CD31-positive staining. (**C**) Clinical expression profile of miR-421 in a Taiwanese OSCC cohort (GSE37991, *left*) and TCGA cohort (GDC Head and Neck Cancer, *right*). (**D**) Kaplan–Meier analysis of overall survival according to miR-421 expression from 239 OSCC patients in TCGA’s cohort (GDC Head and Neck Cancer). Data are presented as mean ± SD; * *p* < 0.05; *** *p* < 0.001.

To further confirm the above findings, the Matrigel plug assay was then used to explore the miR-421 function in angiogenesis in vivo. Matrigel mixed with miR-421 was injected subcutaneously into the flanks of nude mice and analyzed 12 days later. After sacrifice, it was shown that Matrigel plugs mixed with miR-421 (PM421) had more blood vessels and a higher hemoglobin concentration than Matrigel plugs mixed with the scramble control (SC) (Figure 2A). In addition, IHC staining of H&E and CD31 was performed to determine the effect of miR-421 in neovascularization. The results revealed that Matrigel plugs containing miR-421 exhibited more microvessels than those containing the scramble control (Figure 2B). Furthermore, the levels of miR-421 were abnormally elevated in OSCC cells and OSCC patients, consistent with the levels of miR-421 in exosomes released from OSCC cells (Figure 2C and Supplementary Figure S2). Additionally, Kaplan–Meier analysis revealed that miR-421 expression was correlated with poor overall survival in TCGA’s OSCC cohort (*n* = 239) (*p* = 0.043; Figure 2D). Taken together, these findings suggest that OSCC cells expressed a high level of miR-421, which can be secreted by OSCC cells and delivered to HUVECs via exosomes, leading to angiogenesis.

### 2.3. HS2ST1 Is a Target of miR-421 in HUVECs

**Figure 3:**
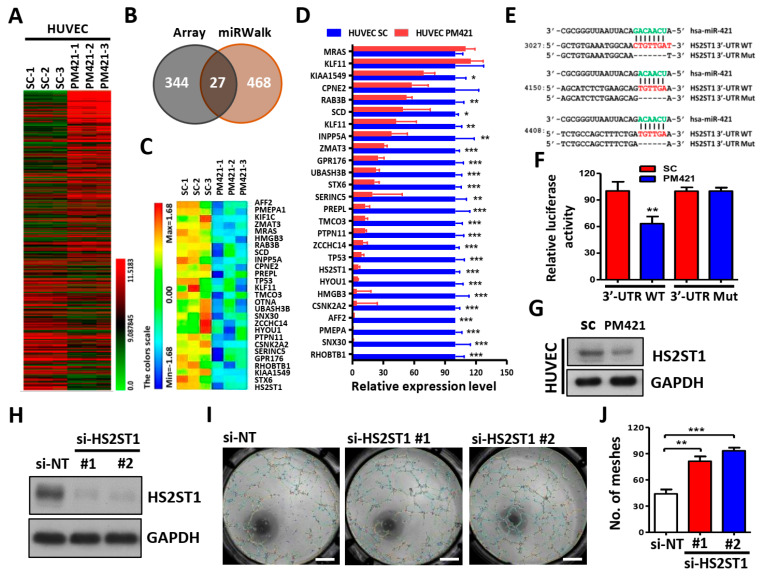
HS2ST1 is a target of miR-421 in HUVECs. (**A**) A heatmap of 371 differentially expressed genes was obtained in HUVECs transfected with miR-421 (PM421, 20 nM) compared to control cells (SC) with three biological replicates using Affymetrix microarrays (ClariomTM S Assay). (**B**) Venn diagram of 27 miR-421-targeted genes based on the intersection of Affymetrix microarray with miRWalk database. (**C**) Heatmap of 27 miR-421-targeted genes. (**D**) Validation of the expression level of 27 genes in miR-421-transfected HUVECs using quantitative real-time PCR. (**E**) Schematic representation of the putative miR-421 binding sequence in the 3′-UTR of HS2ST1 with wild-type form (HS2ST1 3′-UTR WT) and deleted form (HS2ST1 3′-UTR Mut). The matched nucleotides are labeled with green and red color. (**F**) The effect of miR-421 (PM421, 20 nM) on the luciferase activities of the constructs containing the wild-type or mutant-type 3′-UTR in HUVECs. (**G**) Western blot analysis of HS2ST1 in HUVECs following miR-421 (PM4421, 20 nM) or scramble control (SC) transfection for 48 h. GAPDH was used as an internal control. (**H**) Western blot analysis of HS2ST1 protein in HUVECs following transfection of si-HS2ST1 (si-HS2ST1 #1 and #2) or si-non-targeting control (si-NT) for 48 h. (**I**,**J**) Tube formation assay of HUVECs treated with the indicated transfection. Scale bar: 1 mm. Data are presented as mean ± SD; * *p* < 0.05; ** *p* < 0.01; *** *p* < 0.001.

In order to determine the downstream target genes of miR-421 in HUVECs, we performed genome-wide gene expression analysis using miR-421-transfected HUVECs. Compared with control cells, a total of 371 differentially expressed genes were obtained in miR-421-transfected HUVECs (Figure 3A). To search whether these 371 genes are direct target genes of miR-421, we used the miRWalk database (http://mirwalk.umm.uni-heidelberg.de/, accessed on 19 October 2020.) to further compare whether there are binding sites of miR-421 in the 3′-UTR regions of these genes. Through this step, we identified 27 genes that were not only downregulated in miR-421-transfected HUVECs but also contained miR-421 binding sites (Figure 3B,C and Supplementary Table S3). Validation using quantitative real-time PCR revealed that, as expected, most genes were significantly reduced in miR-421-transfected HUVECs (Figure 3D).

Among these genes, we were particularly interested in the HS2ST1 gene because previous studies have shown that HS2ST1 plays an important role in cancer growth, movement, and angiogenesis [28,29,30], but the relevant mechanisms are unclear. Using bioinformatics analysis, we predicted three putative binding sites of miR-421 in the 3′-UTR of HS2ST1 (Figure 3E). We then tested whether miR-421 could directly interact with HS2ST1 through performing a luciferase reporter assay. The relative luciferase activity of the reporter gene containing the HS2ST1 wild-type 3′-UTR was significantly inhibited by transfection with a miR-421 mimic (PM421), but the activity of the reporter gene containing the HS2ST1 mutant 3′-UTR (Mut) showed no significant change (Figure 3F). The protein level of HS2ST1 was also decreased after transfection with PM421 in HUVECs (Figure 3G). Furthermore, we examined the role of HS2ST1 in HUVEC angiogenesis. We found that knockdown of HS2ST1 (si-HS2ST1 #1 and si-HS2ST1 #2) using siRNA technology not only attenuated HS2ST1 protein expression (Figure 3H) but also promoted tube formation in HUVECs (Figure 3I,J). These data suggested that HS2ST1 acts as a direct target gene of miR-421 and is involved in HUVEC angiogenesis.

### 2.4. HS2ST1 Modulates VEGF-Mediated Activation of ERK and AKT in HUVECs

**Figure 4:**
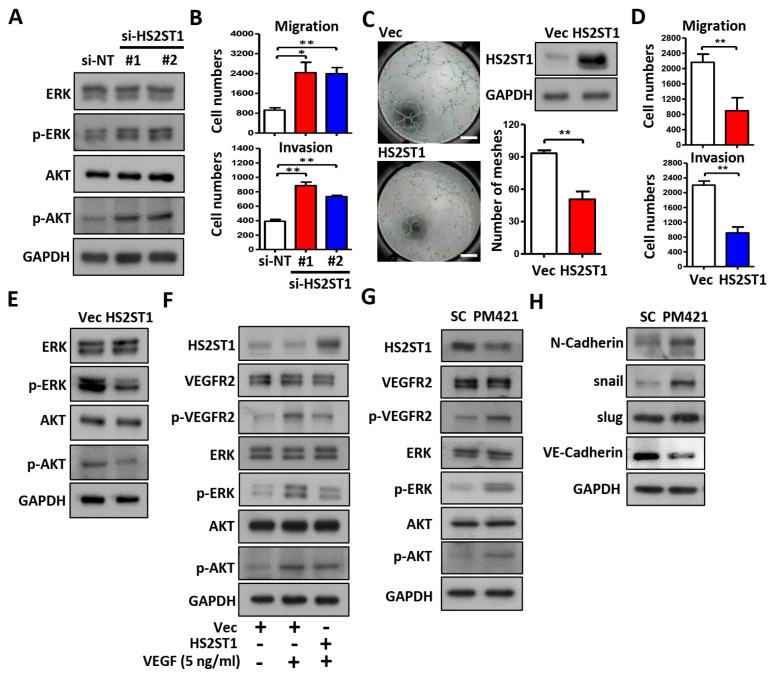
HS2ST1 blocks VEGF-induced tube formation. (**A**) Western blot analysis of ERK, phosphor-ERK (p-ERK), AKT, and phosphor-AKT (p-AKT) after transfecting the si-HS2ST1 (si-HS2ST1 #1 and #2) or si-non-targeting control (si-NT) for 48 h. GAPDH was used as an internal control. (**B**) Transwell migration and invasion assay of HUVECs following transfection of siRNA for 24 h. (**C**) Tube formation assay of HUVECs after transfection with HS2ST1 expression vector or control vector (Vec). Western blot analysis showed the HS2ST1 protein level (*right upper*). The mean number of meshes was statistically analyzed (*right bottom*). Scale bar: 1 mm. (**D**) Transwell migration and invasion assay of HUVECs following transfection of HS2ST1 expression vector or control vector (Vec) for 24 h. (**E**–**H**) Western blot analysis of ERK, phosphor-ERK (p-ERK), AKT, phosphor-AKT (p-AKT), HS2ST1, VEGFR2, phosphor-VEGFR2 (p-VEGFR2), N-Cadherin, snail, slug, and VE-Cadherin in HUVECs treated with the indicated treatment and transfection. GAPDH was used as an internal control. Data are presented as mean ± SD; * *p* < 0.05; ** *p* < 0.01.

We next tested whether HS2ST1 could affect angiogenesis-related ERK and AKT signaling. The phosphorylation of ERK and AKT was increased in HS2ST1 knockdown HUVECs compared with control cells (si-NT) (Figure 4A). In addition, knockdown of HS2ST1 also resulted in enhanced migration and invasion abilities of HUVECs (Figure 4B). In contrast, HS2ST1 overexpression showed decreased migration, invasion, and tube formation (Figure 4C,D) in HUVECs. The phosphorylation of ERK and AKT was decreased in HS2ST1-overexpressing HUVECs compared with control cells (NT) (Figure 4E). These results suggested an important role of HS2ST1 in mediating angiogenesis-related signaling pathways. Previous studies have reported that 2-O-sulfated HS oligosaccharides by HS2ST1 can block basic fibroblast growth factor (FGF-2) binding to its receptor and modulate FGF-2-mediated MAPK signaling [14,31]. Given these findings, we hypothesized that HS2ST1 may be involved in the VEGF-induced ERK and AKT activation in HUVECs. To test this hypothesis, we examined the phosphorylation level of VEGFR2, ERK, and AKT in the presence of VEGF (5 ng/mL). As expected, HS2ST1 overexpression potently suppressed the VEGF-induced phosphorylation of VEGFR2, ERK, and AKT in HUVECs (Figure 4F). We then asked whether miR-421 could regulate the activities of VEGFR2, ERK, and AKT. We observed that miR-421 (PM421) overexpression not only decreased the expression of HS2ST1 but also increased the phosphorylation levels of VEGFR2, ERK, and AKT (Figure 4G). In addition, we also found that overexpression of miR-421 increased the expression of mesenchymal markers, such as N-cadherin, snail, and slug and, conversely, decreased the expression of epithelial markers, such as VE-cadherin (Figure 4H). Overall, these data demonstrated that the miR-421/HS2ST1 axis modulates the VEGF/VEGFR2-mediated activation of ERK and AKT, indicating a shifting trend for the EMT phenotype.

### 2.5. Exosomal miR-421 Promotes Tube Formation by Targeting HS2ST1

**Figure 5:**
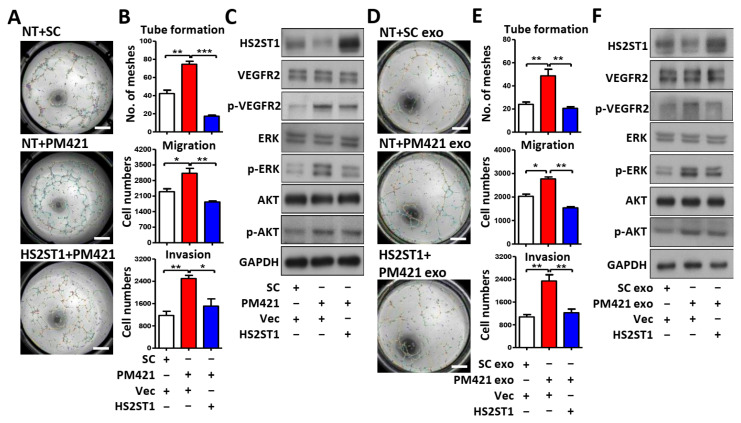
Exosomal miR-421 promotes tube formation by targeting HS2ST1 in HUVECs. (**A**,**D**) Images of the tube formation assay of HUVECs treated with the indicated transfection. Scale bar: 1 mm. (**B**,**E**) Transwell migration, invasion, and tube formation assay of HUVECs treated with the indicated transfection. Data are presented as mean ± SD; * *p* < 0.05; ** *p* < 0.01; *** *p* < 0.001. (**C**,**F**) Western blot analysis of HS2ST1, VEGFR2, phosphor-VEGFR2 (p-VEGFR2), ERK, phosphor-ERK (p-ERK), AKT, and phosphor-AKT (p-AKT) in HUVECs treated with the indicated transfection. GAPDH was used as an internal control.

Thus far, we have demonstrated that HS2ST1 is the direct target of miR-421, but whether miR-421 induces tube formation through HS2ST1 remains unclear. To determine whether miR-421-induced tube formation is mediated by HS2ST1, we co-transfected HUVECs with the miR-421 mimic (PM421) and an HS2ST1 expression plasmid that did not contain the 3′-UTR of HS2ST1. We observed that miR-421-induced HUVEC migration, invasion, tube formation, and phosphorylation of VEGFR2, ERK, and AKT were inhibited by HS2ST1 overexpression (Figure 5A–C). We repeated the above experiments by replacing miR-421 with miR-421-enriched exosomes isolated from OEC-M1 cells. Consistent with the above observation, HS2ST1 overexpression also suppressed the exosomal–miR-421-induced HUVEC migration, invasion, tube formation, and phosphorylation of VEGFR2, ERK, and AKT (Figure 5D–F). These results indicated that exosomal miR-421 promotes HUVEC tube formation and modulates angiogenetic signaling, mainly through HS2ST1 downregulation.

### 2.6. LncRNA MEG3 Inhibits Tube Formation via Targeting miR-421

**Figure 6:**
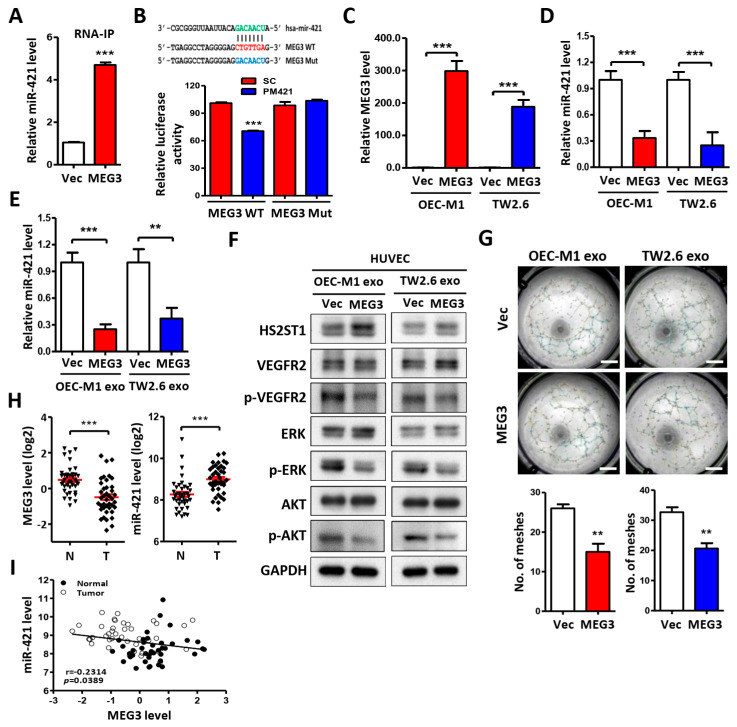
LncRNA MEG3 inhibits tube formation via targeting miR-421. (**A**) Following MS2 RNA-immunoprecipitation in OEC-M1 cells, RNA was extracted by Trizol–chloroform and precipitated by isopropanol plus glycogen at −20 °C. The level of miR-421 was measured by qRT-PCR in the pCDH-MEG3-MS2 group (MEG3) compared with the pCDH-MS2 control vector group (Vec). (**B**) *Upper*, schematic representation of the putative miR-421 binding sequence in the lncRNA MEG3 with wild-type form (MEG3 WT) and mutant form (MEG3 Mut). The matched nucleotides are labeled with green and red color. The mutant nucleotides are labeled with blue color. *Lower*, the effect of miR-421 (PM421, 20 nM) on the luciferase activities of the constructs containing the wild-type or mutant-type MEG3 in OEC-M1 cells. (**C**,**D**) Relative expression of MEG3 and miR-421 in OEC-M1 and TW2.6 cells after transfection of MEG3 expression vector or control vector (Vec). (**E**–**G**) Relative miR-421 levels, Western blot analysis, and tube formation assay in HUVECs after incubation with exosomes derived from OEC-M1 and TW2.6 cells, which were transfected with MEG3 expression vector or control vector (Vec). Scale bar: 1 mm. (**H**) MEG3 or miR-421 expression level by qRT-PCR in 40 OSCC tumors (T) compared with their own adjacent normal tissues (N). Expression levels are expressed as the log_2_ ratios. The red line represents the mean ± SD. (**I**) Correlation analysis of miR-421 and MEG3 in human OSCC patients. Data are presented as mean ± SD; ** *p* < 0.01; *** *p* < 0.001.

Recently, lncRNA MEG3 was reported to act as a competing endogenous RNA (ceRNA) to regulate tumorigenesis by targeting miRNAs [32]. Therefore, we were very interested in exploring whether lncRNA MEG3 competitively binds and absorbs miR-421 as a ceRNA in OSCC cells. Using RNA immunoprecipitation (RNA-IP), we attempted to pull down endogenous miRNA bound to lncRNA MEG3. The results showed that MEG3 RNA-IP was significantly enriched for miR-421 in OEC-M1 cells compared with the MS2 control vector (Figure 6A). Furthermore, the transfection of wild-type (WT) MEG3 and PM421 reduced luciferase activity compared to the group transfected with the MEG3 mutant (Mut) and miR-421 mimic (Figure 6B), indicating that lncRNA MEG3 could suppress the expression level of miR-421 through direct binding. We then asked whether MEG3 affects HUVEC angiogenesis through exosomal miR-421. We observed that MEG3 overexpression (Figure 6C) not only reduced the level of cellular miR-421 (Figure 6D) but also decreased the level of exosomal miR-421 (Figure 6E) in OSCC cells. In addition, HUVECs treated with exosomes derived from MEG3-overexpressing OSCC cells not only significantly increased the HS2ST1 protein level but also decreased VEGFR2, ERK, and AKT phosphorylation (Figure 6F) and the tube formation of HUVECs (Figure 6G). To confirm our findings, we also examined the expression profiles of lncRNA MEG3 and miR-421 in OSCC patients. The data showed that the MEG3 levels were significantly downregulated and the miR-421 levels were significantly upregulated in tumors (T) compared with corresponding normal samples (N) (*p* < 0.0001) (Figure 6H). Moreover, the expression level of lncRNA MEG3 was significantly negatively correlated with the expression level of miR-421 (Figure 6I). Taken together, these results indicate that the downregulation of lncRNA MEG3 in OSCC cells can stimulate angiogenesis by regulating miR-421 expression, promoting exosomal miR-421 release, and targeting HUVECs.

### 2.7. Blocking Exosome Uptake Inhibits Tube Formation in HUVECs

**Figure 7:**
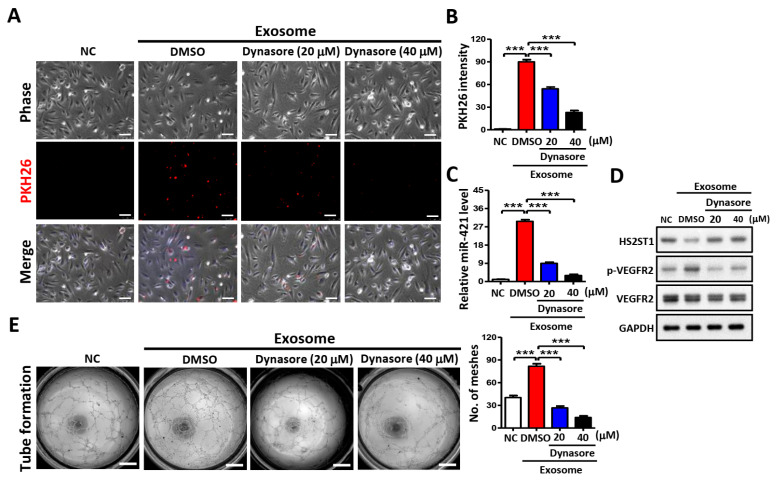
Effects of exosome uptake in HUVECs. (**A**) Images of exosome uptake at 2 h after dynasore treatment in HUVECs under a confocal microscope. Exosomes isolated from OEC-M1 were stained with PKH26 and added to the culture medium of dynasore-treated (20 or 40 μM) or untreated (Mock) HUVECs. As a negative control (NC), HUVECs were incubated with DMSO only. Scale bar: 50 µm. (**B**) Exosome uptake was assessed by measuring PKH26 fluorescence intensity using a FACSCalibur cytometer. (**C**) Effect of exosome uptake on miR-421 levels after dynasore treatment in HUVECs using qRT-PCR. (**D**) Effect of exosome uptake on protein levels after dynasore treatment in HUVECs using Western blot analysis. GAPDH was used as an internal control. (**E**) Effect of exosome uptake on tube formation at 6 h after dynasore treatment in HUVECs. Scale bar: 1 mm. Data are presented as mean ± SD; *** *p* < 0.001.

To identify the tube formation process that contributes to exosome internalization, HUVECs were treated with OSCC cell-derived exosomes and dynasore, an inhibitor of exosome internalization [33]. We found that the red fluorescence was decreased in HUVECs, depending on the dynasore concentration (Figure 7A). Dynasore significantly blocked 50% of exosome uptake (20 μM) and 91% of exosome uptake (40 μM) compared with that in DMSO control HUVECs (Figure 7B). Dynasore not only inhibited exosome uptake but also decreased miR-421 levels in endothelial cells (Figure 7C), which, in turn, increased HS2ST1 protein expression, decreased VEGFR2 phosphorylation (Figure 7D), and ultimately suppressed tube formation in HUVECs (Figure 7E). These results suggest that exosomes released by OSCC cells can act as angiogenesis promoters, and inhibiting exosome uptake by HUVECs may be a potential therapeutic approach for cancer treatment.

## 3. Discussion

**Figure 8:**
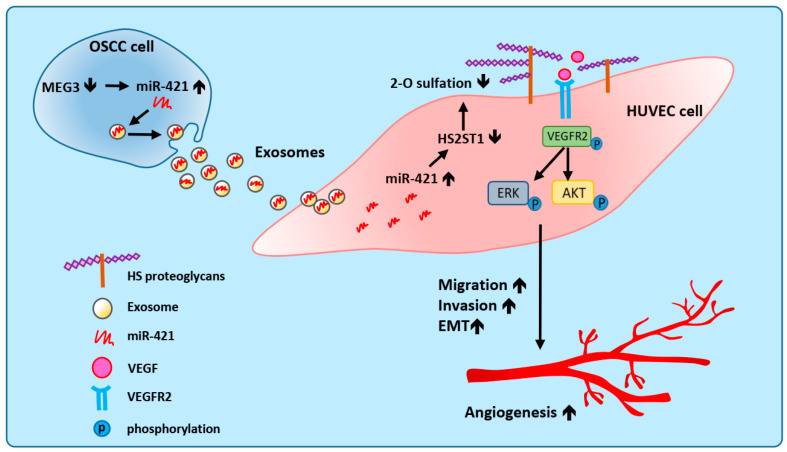
Proposed model illustrating that exosomal miR-421 modulates angiogenesis in OSCC. Downregulated lncRNA MEG3 increases the amount of miR-421, which is then packaged in OSCC-derived exosomes and secreted into the tumor microenvironment. MiR-421-enriched exosomes are taken up by HUVECs to induce angiogenesis through targeting HS2ST1. Downregulation of HS2ST1 leads to decreased 2-O-sulfation of HSPG, thereby facilitating VEGF binding to VEGFR2, which then promotes the EMT, migration, invasion, and angiogenic potential of HUVECs.

In this study, we showed that OSCC-derived exosomal miR-421 can target endothelial cells to increase angiogenesis by targeting HS2ST1 via VEGFR2 and downstream signaling pathways. Furthermore, we also found that miR-421 was negatively regulated by lncRNA MEG3, which acts as a ceRNA in OSCC (Figure 8). Therefore, our study not only suggests that blocking the transmission of pro-angiogenic miRNAs in exosomes might be an effective antiangiogenic therapeutic approach for OSCC but also provides insights about the regulation and functionality of lncRNA MEG3.

LncRNA MEG3 is located in the imprinted DLK1–MEG3 locus on the human chromosome 14q32 region [34]. An increasing number of studies have shown that lncRNA MEG3 is hypermethylated and downregulated in various cancer tissues and cancer cell lines, playing the role of a tumor suppressor gene, and that it is a good biomarker for cancer diagnosis and prognosis [35,36]. In terms of the role of ceRNA, lncRNA MEG3 can bind different miRNAs like a “sponge” and contribute to the regulatory network of miRNAs [37,38,39]. Consistent with these findings, we also showed that MEG3 exerted its regulatory role in oral cancer by interacting with miR-421, with several novel observations: (1) MEG3 restoration not only reduced the levels of miR-421 in OSCC cells but also resulted in decreased miR-421 levels in OSCC-derived exosomes. (2) OSCC-derived exosomes from MEG3 restoration resulted in reduced VEGFR2, ERK, and AKT activities, leading to reduced tube formation in HUVECs. Our study shows that downregulated MEG3 can increase the expression of miR-421, which is then packaged in OSCC cell-derived exosomes and secreted into the extracellular microenvironment to target vascular endothelial cells and induce angiogenesis. To our knowledge, this is the first study to show that lncRNA MEG3 can affect exosomal miRNA secretion from OSCC and cause angiogenesis in the tumor microenvironment.

Recently, exosomes have been receiving enormous attention in both basic and translational medicine [40]. This is mainly because exosomes derived from cancer cells can change the behavior of target cells in the tumor microenvironment, and, more importantly, exosomes have shown utility in the diagnosis and treatment of various cancers [41]. Zhang et al. reported that the treatment of esophageal squamous cell carcinoma cells with the exosome inhibitor SW4869 largely attenuated the migration and tube formation of HUVECs [42]. Collectively, these results demonstrate that cancer cell-derived exosomes exert an angiogenic effect and suggest that exosomes are, indeed, potential targets for cancer therapy, whether by blocking the generation of exosomes in cancer cells or by inhibiting exosome internalization in HUVECs. Many in vitro studies and pre-clinical in vivo studies indicate that lots of compounds have the ability to block the formation and release of exosomes (such as imipramine, simvastatin, DPTIP, and Calpeptin), which may prove useful for interfering in the progression of certain types of cancers [43]. These inhibitors affect different stages of exosome biogenesis and trafficking, and the disruption of cell-to-cell communication from cancer cell-derived exosomes appears to provide an exciting new approach to cancer therapy [44]. Currently, based on research results and clinical trials, exosomes are considered immunotherapeutic vaccines for various cancers [40]. Dendritic cell (DC)-derived exosomes have been found to activate natural killer cells and antigen-specific cytotoxic T lymphocytes in the treatment of patients [45]. Thus, using natural sources of exosomes from immune cells to suppress tumor growth represents an important strategy for cancer therapy.

Emerging evidence suggests that exosomal cargo, especially miRNA, is valuable for predicting disease progression and prognosis [46]. In addition to avoiding RNase-mediated degradation, another advantage of packaging miRNAs into cellular exosomes is that they can be easily taken up by neighboring cells or carried to distant sites, thus potentially inducing phenotypic changes in recipient cells. Since oral cancer cell-derived exosomes can enhance endothelial cell migration and tube formation, the purpose of this study was to identify and characterize selective exosomal miRNA profiles and speculate on their potential targets involved in angiogenesis. Our study showed that a high expression of miR-421 in oral cancer cell and OSCC patients is clinically associated with poor patient survival, suggesting that miR-421 may be a useful biomarker for OSCC prognosis. Additionally, we found that HS2ST1 expression was lower in HUVECs treated with exosomal miR-421. The restoration of HS2ST1 reversed exosomal miR-421-induced HUVEC tube formation, suggesting that HS2ST1 contributes to miR-421-mediated angiogenesis. The aberrant expression of HS2ST1 is frequently observed in tumors, suggesting that HS2ST1 plays an important role in tumor progression. For example, the upregulation of HS2ST1 was associated with reduced invasive ability by attenuating FGF-2-induced MAPK activation in breast cancer cells [14]. In addition, the overexpression of HS2ST1 can decrease the stemness phenotype in the triple-negative MDA-MB-231 breast cancer cell line, possibly by regulating the notch and Wnt signaling pathways [47]. In this report, we demonstrate a novel role for HS2ST1 in regulating tube formation in vascular endothelial cells through VEGFR2-mediated MAPK signaling. Importantly, HS2ST1 overexpression reduced the VEGF-induced phosphorylation of VEGFR2, ERK, and AKT, suggesting that HS 2-O-sulfation by HS2ST1 interferes with the binding of VEGF to its receptor, VEGFR2. The mechanism by which HS2ST1 inhibits VEGF signaling is unclear. However, previous studies have shown that 2-O-sulfated HS oligosaccharides are highly negatively charged, interacting with positively charged ligand proteins, thereby blocking ligand–receptor binding [31]. Another possible reason is that the HS sulfated by HS2ST1 may induce a conformational change in HS that hinders the binding of VEGF and VEGFR2. Therefore, whether it is the charge change or the configuration change that blocks ligand engagement needs further investigation.

## 4. Materials and Methods

### 4.1. Cell Culture

OSCC cell lines (OEC-M1 and TW2.6) were cultured as previously described [48]. Transformed normal human keratinocytes (OKF4/hTERT) were obtained from Rheinwald lab and cultured in an oral keratinocyte medium (OKM; ScienCell Research Laboratories, Carlsbad, CA, USA) according to the manufacturer’s instructions. HUVECs were cultured in M199 medium (Gibco, Grand Island, NY, USA) supplemented with 500 units/mL of heparin (Sigma–Aldrich, St. Louis, MO, USA) and 0.03 mg/mL endothelial cell growth supplement (Sigma–Aldrich). All cells were verified based on cell morphology and growth features and cultured at 37 °C in 10% fetal bovine serum (FBS, Kibbutz Beit Haemek, Israel) within 2 months of resuscitation from the frozen stock.

### 4.2. Exosome Purification, Characterization, and Quantification

Exosomes from the culture medium were purified via the ultracentrifugation method. Briefly, the cell culture medium was replaced with a serum-free medium for 48 h. The collected culture medium was centrifuged at 300× *g* for 10 min to remove cellular debris. The supernatant was then centrifuged at 1200× *g* for 20 min and 10,000× *g* for 30 min to remove large vesicles. Finally, the supernatant was ultracentrifuged at 110,000× *g* for 2 h to collect exosome pellets using a Beckman Optima XL-90 ultracentrifuge (GMI Inc., Ramsey, MN, USA). The obtained exosome pellets were lastly washed and resuspended in PBS.

The morphology of exosome pellets was examined under a Hitachi H-7650 transmission electron microscope (TEM) (Hitachi Ltd., Tokyo, Japan). Briefly, 5 μL of exosomes in PBS drops was loaded onto carbon-coated copper grids and left at room temperature for 10 min. The exosomes on the copper mesh were washed with sterile distilled water and then incubated with 1% phosphotungstic acid for 5 min. We allowed the samples to dry under the lamp for 10 min before viewing on the TEM. To examine the particle size and quantity of exosomes, dynamic light scattering (DLS) was performed using a Nano Zetasizer (Malvern Instruments Inc., Westborough, MA, USA) according to the operating instructions.

The BCA kit was used to measure the protein concentration of exosomes. Thus, 25 μg of exosomes was used for each experiment. The exosome-specific surface proteins CD9 (GTX100912, GeneTex, Irvine, CA, USA) and CD81 (GTX101766, GeneTex) were detected using Western blotting.

### 4.3. Tube Formation Assay

For the tube formation assay, a precooled 96-well plate was coated with 10 μL of Matrigel (BD Biosciences, Franklin Lakes, NJ, USA) in each well and incubated at room temperature for 1 h. A total of 3500 HUVECs in a cell suspension were added to each well and incubated for 6 h, and the tubes that formed were imaged and counted with the image J software (Ver.1.52a) package. The mean number of meshes was statistically analyzed and used as the angiogenesis index.

### 4.4. Cell Migration and Invasion Assays

The 24-well Fluoro-Blok inserts (#351152, BD Biosciences, Franklin Lakes, NJ, USA) were utilized to measure the invasion and migration ability of HUVECs. Therefore, 3 × 10^4^ cells were seeded in the upper chamber coated with (for invasion assay) or without (for migration assay) Matrigel (BD Biosciences) in a culture medium containing 10% NuSerum. Following an 8-h incubation for migration assays and a 24-h incubation for invasion assays, the cells that had crossed the Fluoro-Blok membrane were fixed with 95% methanol and stained with propidium iodide. Fluorescent images were then counted using the image J software (Ver.1.52a) package.

### 4.5. RNA Extraction, Reverse Transcription, and Quantitative PCR (q-PCR)

TRIzol reagent (Life Technologies, Gaithersburg, MD, USA) was used to purify the total RNA and exosomes based on the manufacturer’s protocol. The RNA concentration was determined by a NanoDrop ND-1000 spectrophotometer (Thermo Fisher Scientific, Wilmington, DE, USA). For mRNA and lncRNA analyses, the cDNA was synthesized using random hexamer primers and SuperScript III reverse transcriptase (Invitrogen, Carlsbad, CA, USA). For miRNA analysis, the cDNA was synthesized using specific stem-loop RT primers and the TaqMan MicroRNA Reverse Transcription Kit (Applied Biosystems, Carlsbad, CA, USA). q-PCR analysis was performed using Omics Green EvaGreen q-PCR Master Mix (OMICS Biotechnology, New Taipei City, Taiwan) to detect gene expression levels and using the QuantiTect SYBR Green PCR System (Qiagen, Hilden, Germany) to detect miRNA expression levels according to the manufacturer’s instructions on the ABI StepOnePlus Real-time PCR system (Applied Biosystems). Bdi-miR-159-5p was used as the spike-in control for exosomal miRNA analysis, while GAPDH and RUN44 were used as the internal controls for the detection of mRNA and miRNA in cells. All primers used for this study are listed in Supplementary Table S4.

### 4.6. Exosomal miRNA Profiling

Exosomal miRNA isolated from OKF4/hTERT, OEC-M1, and TW2.6 cells was extracted using TRIzol reagent (Life Technologies). The miRNA RNA purity, concentration, and integrity were determined using a NanoDrop ND-1000 spectrophotometer (Thermo Fisher Scientific, Wilmington, DE, USA) and Agilent 2100 Bioanalyzer (Agilent Technologies, Santa Clara, CA, USA). Exosomal miRNA profiling was performed using an Affymetrix, GeneChip^TM^ miRNA 3.0 array (Thermo Fisher Scientific), according to the manufacturer’s protocol.

### 4.7. In Vivo Matrigel Plug Assay

A total of 400 μL of growth factor-reduced Matrigel HC (#354262, Corning Inc., Corning, NY, USA), containing 15 U of heparin (#H3393, Sigma–Aldrich) and 100 μL of miR-421 mimics (PM421) or scramble control (SC), was injected into the right flank of 6-week-old male nude mice (BALB/cAmM.CgFoxn1nu/CrlNarl), 4 mice per group. The plug was removed after 12 days, and half of the plug was formalin-fixed and sliced for immunohistochemistry [49], while the other half of the plug was used for hemoglobin analysis. The presence of the blood endothelial cell-specific marker was assessed using a CD31 antibody (#3528, Cell signaling, Danvers, MA, USA). The hemoglobin concentration was measured using a hemoglobin kit (#EIAHGBC, Invitrogen, Carlsbad, CA, USA) following the manufacturer’s protocol as an indicator of the angiogenic index.

### 4.8. Clinical Samples and Microarray Profiling

A total of 40 paired OSCC tissues, including tumor and their adjacent non-tumorous tissues, were obtained from the National Cheng Kung University Hospital (Tainan, Taiwan), as previously described [48]. The research protocol was reviewed and approved by the Research Ethics Committee of the National Health Research Institutes (No: EC1091104-E) and the Institutional Human Experiment and Ethics Committee of National Cheng Kung University Hospital (HR-97-100). These matched pairs of oral tumor/adjacent normal (T/N) tissues were prepared for microarray analysis using the whole-genome DASL HumanRef-8-v3 chip and the Human-2v MicroRNA Expression BeadChips (Illumina Inc., San Diego, CA, USA). These microarray profiling data are deposited in the Gene Expression Omnibus (GEO) under accession number GSE37991 for gene expression and GSE45238 for miRNA expression. The Kaplan–Meier analysis of overall survival according to miR-421 expression from 239 OSCC patients was analyzed from The Cancer Genome Atlas (TCGA) HNSCC cohort (GDC Head and Neck Cancer).

### 4.9. Plasmids, Transfection, and Virus Infection Assays

To construct the dual-luciferase reporter vectors, the entire 3′-untranslated region (3′-UTR) of the HS2ST1 fragment and the entire MEG3 gene, containing the target sequences of miR-421, were PCR amplified and cloned into the pmirGLO vector (Promega, Madison, WI, USA) according to the manufacturer’s instructions. The miR-421 binding site mutation vectors were also constructed by using a Site-Directed Mutagenesis Kit (Stratagene, La Jolla, CA, USA), and all the constructs were verified through DNA sequencing.

For the transfection of the plasmids, cells were transfected with 2 μg of plasmid using Lipofectamine 2000 (Invitrogen) according to the manufacturer’s protocol. The miRNA mimics (PM) and scramble control (SC) were obtained from Thermo Fisher Scientific; the small interfering RNAs (siRNAs) of HS2ST1 (si-HS2ST1 #1, #2) and si-non-targeting control (si-NT) were purchased from Horizon Discovery (Cambridge, UK). The nucleotide transfection was performed using Lipofectamine RNAiMAX (Thermo Fisher Scientific) according to the manufacturer’s instructions.

The lentiviral vectors expressing HS2ST1 or the control (NT) were transfected into the packaging cell line 293FT, along with the pMD.G and pCMV△R8.91 plasmids, using the Polyjet transfection reagent (SignaGen Lab, Ijamsville, MA, USA), and the lentiviruses were collected after 48 h to infect HUVECs.

### 4.10. Luciferase Reporter Assay

Cells were co-transfected with the reporter vector containing the 3′-UTR of HS2ST1 or the MEG3 sequence and miR-421 mimics (PM421, 20 nM) or scramble control (SC, 20 nM). After 48 h, firefly and Renilla luciferase activities were detected using the Dual Luciferase Reporter Assay System (Promega, Madison, WI, USA) on the Orion L luminometer (Berthold, GmbH, Pforzheim, Germany) according to the manufacturer’s protocol.

### 4.11. Protein Extraction and Western Blotting Analysis

Protein extraction and Western blotting were performed as previously described [50]. The primary antibodies used in the present study included HS2ST1 (sc-376530, Santa Cruz Biotechnology, Santa Cruz, CA, USA), CK18 (sc-6259, Santa Cruz Biotechnology), ERK (#9102, Cell Signaling, Beverly, MA, USA), phosphor-ERK (p-ERK, #9101, Cell Signaling), AKT (#9272, Cell Signaling), phosphor-AKT (p-AKT, #9271, Cell Signaling), VEGFR2 (#24792, Cell Signaling), and phosphor-VEGFR2 (p-VEGFR2, #2478, Cell Signaling). GAPDH (GTX100118, GeneTex) was used as an internal control. Signals from HRP-conjugated secondary antibodies were visualized using the enhanced chemiluminescence (ECL) detection system (PerkinElmer, Waltham, MA, USA), and the chemiluminescence was exposed onto Kodak X-Omat film (Kodak, Chalon/Paris, France).

### 4.12. RNA Immunoprecipitation (RNA-IP)

RNA-IP was performed as previously described with modification [51]. Briefly, cells were infected with virus particles that contained pCDH-HA-2xMCP-SBP, pCDH-24xMS2, or pCDH-MEG3-24xMS2 plasmids for 48 h. Then, the cells were lysed using RIPA extraction buffer containing an RNase inhibitor and protease inhibitor cocktail (Sigma–Aldrich, Inc., St. Louis, MO, USA). The protein concentration was then determined using the BCA assay kit (Thermo Fisher Scientific) with bovine serum albumin as the standard. Before immunoprecipitation, a Streptavidin-Sepharose bead slurry (GE Healthcare, Chicago, IL, USA) was pre-swollen in NT2 buffer (50 mM Tris-HCl, pH 7.4, 150 mM NaCl, 1 mM MgCl_2_, 0.05% NP-40) supplemented with 20 mM DTT, 20 mM EDTA, and 200 U RNase inhibitor. Equal amounts of protein lysates were subjected to immunoprecipitation overnight by mixing with Streptavidin-Sepharose beads at 4 °C. Pellet beads were then washed with ice-cold NT2 buffer plus 0.5 M Urea (Mallinckrodt, Phillipsburg, NJ, USA) 4–5 times. To isolate RNA from immunoprecipitated pellets, phenol-chloroform-isoamyl alcohol with glycogen was added to the beads at −20 °C overnight. The precipitated RNA was resuspended in a volume suitable for subsequent assays.

### 4.13. Fluorescence Analysis and Exosome Uptake Assay

For exosome uptake experiments, HUVECs were pretreated with an endocytosis inhibitor (dynasore) or dimethyl sulfoxide (DMSO) solvent for 6 h. Then, HUVECs were washed with PBS and incubated with PKH26 (Sigma–Aldrich)-labeled exosomes (25 μg) isolated from OEC-M1 cells at 37 °C for 2 h. As a negative control, HUVECs were incubated with DMSO only. The uptake of exosomes in HUVECs was photographed under a TCS SP5 II Confocal Microscope (Leica, Wetzlar, Germany). Exosome uptake was also assessed by measuring the PKH26 fluorescence intensity using a FACSCalibur cytometer equipped with CellQuest™ Pro software, Ver.4.1 (BD Biosciences).

### 4.14. Statistical Analysis

All statistical analyses were performed using GraphPad Prism 5 software Ver.5.01 (San Diego, CA, USA), and the results are expressed as mean ± standard deviation (SD). Differences between groups were analyzed using two-tailed Student’s *t*-test. Correlations between data pairs were performed by parametric Pearson Spearman correlation analysis. Values of *p* < 0.05 were considered statistically significant.

## 5. Conclusions

Our results demonstrated a novel mechanism of MEG3 whereby downregulation of this lncRNA increases the amount of OSCC-derived exosomal miR-421 that is taken up by vascular endothelial cells to induce angiogenesis by targeting HS2ST1 and additional signaling pathways. Our study is the first to highlight the critical role of exosomal miR-421 in an HS modifier enzyme, HS2ST1, in HUVECs. These findings further suggest that targeting the lncRNA MEG3/exosomal miR-421/HS2ST1 axis may be an effective strategy for the treatment of OSCC.

## Data Availability

The dataset supporting the conclusions of this article is included within the article.

## Supplementary Materials

The following supporting information can be downloaded at: https://www.mdpi.com/article/10.3390/ijms25147576/s1.
