# Supplementary material for: MEG3-Mediated Oral Squamous-Cell-Carcinoma-Derived Exosomal miR-421 Activates Angiogenesis by Targeting HS2ST1 in Vascular Endothelial Cells
Source: Int J Mol Sci. 2024 Jul 10;25(14):7576. doi: 10.3390/ijms25147576 (PMC11277508; doi:10.3390/ijms25147576)
Supplement: Supplementary file 1 [file ijms-25-07576-s001.zip › ijms-2970281-supplementary.pdf]

Supplementary Data

MEG3-mdeiated oral squamous cell carcinoma-derived exosomal miR-421 activates angiogenesis by targeting HS2ST1 in vascular endothelial cells

Chia-Yun Huang, Sung-Tau Chou, Yuan-Ming Hsu, Wan-Ju Chao, Guan-Hsun Wu, Jenn-Ren Hsiao, Horng-Dar Wang, Shine-Gwo Shiah

Supplementary Table S1. List of 181 exosomal miRNA candidates.

miRNA	OEC-M1/OKF4-hTERT exosom array (Ratio)	TW2.6/OKF4-hTERT exosom array (Ratio)
hsa-let-7g-5p	38.251	10.454
hsa-miR-100-5p	34.855	65.913
hsa-miR-103a-3p	4.775	4.051
hsa-miR-106a-5p	4.866	2.427
hsa-miR-106b-3p	29.482	16.579
hsa-miR-107	4.861	4.588
hsa-miR-1182	5.179	4.547
hsa-miR-1225-5p	10.299	21.023
hsa-miR-122-5p	88.766	110.564
hsa-miR-1229-5p	10.628	15.572
hsa-miR-1231	22.166	10.149
hsa-miR-1233-5p	6.859	8.841
hsa-miR-1236-5p	2.585	3.160
hsa-miR-1246	21.374	10.498
hsa-miR-1247-3p	17.463	25.951
hsa-miR-125a-3p	12.302	6.029
hsa-miR-126-3p	27.891	11.145
hsa-miR-1273d	5.327	7.146
hsa-miR-1273f	14.620	14.594
hsa-miR-1273h-5p	6.978	10.288
hsa-miR-1275	37.807	47.102
hsa-miR-1301-3p	11.771	7.179
hsa-miR-130a-3p	36.859	83.646
hsa-miR-130b-3p	37.953	74.923
hsa-miR-140-3p	16.091	5.898
hsa-miR-141-3p	31.487	6.066
hsa-miR-150-3p	15.560	14.404
hsa-miR-151b	25.949	12.069
hsa-miR-15b-5p	56.238	55.700
hsa-miR-17-5p	5.680	2.856
hsa-miR-183-5p	39.878	19.095
hsa-miR-185-3p	5.379	14.516
hsa-miR-185-5p	67.956	73.569
hsa-miR-18a-5p	183.090	56.841

hsa-miR-18b-5p	26.527	6.450
hsa-miR-1909-3p	14.824	25.000
hsa-miR-194-5p	54.322	7.029
hsa-miR-196a-5p	105.880	44.198
hsa-miR-197-5p	32.075	26.625
hsa-miR-200a-3p	25.519	5.057
hsa-miR-200b-5p	33.918	7.729
hsa-miR-204-3p	43.970	8.585
hsa-miR-20b-5p	60.133	17.688
hsa-miR-21-5p	28.707	6.015
hsa-miR-2115-5p	0.156	0.113
hsa-miR-2392	11.934	15.360
hsa-miR-25-3p	123.471	73.624
hsa-miR-27b-3p	141.455	57.454
hsa-miR-28-5p	21.308	10.712
hsa-miR-296-3p	7.515	7.634
hsa-miR-30a-5p	29.903	19.467
hsa-miR-30d-5p	28.036	15.349
hsa-miR-3147	4.802	8.003
hsa-miR-3154	8.956	14.250
hsa-miR-3162-5p	30.564	50.952
hsa-miR-3175	66.096	54.965
hsa-miR-3180	11.046	12.080
hsa-miR-3180-3p	29.699	30.093
hsa-miR-3185	9.429	8.297
hsa-miR-3187-3p	4.822	4.156
hsa-miR-320e	40.335	8.804
hsa-miR-32-3p	4.217	16.420
hsa-miR-324-3p	13.645	6.409
hsa-miR-330-3p	4.911	4.908
hsa-miR-361-5p	16.560	10.893
hsa-miR-3648	7.999	10.282
hsa-miR-3651	55.852	19.577
hsa-miR-3652	3.612	12.325
hsa-miR-365a-5p	4.357	2.758
hsa-miR-3663-3p	11.208	25.375
hsa-miR-3679-5p	23.087	19.489
hsa-miR-378c	10.640	9.262

hsa-miR-378h	0.108	0.123
hsa-miR-3937	11.079	31.057
hsa-miR-421	14.693	11.292
hsa-miR-423-5p	41.319	20.785
hsa-miR-4253	18.622	11.695
hsa-miR-425-5p	51.086	12.335
hsa-miR-4298	14.113	13.632
hsa-miR-4322	4.857	11.745
hsa-miR-4417	34.841	68.459
hsa-miR-4430	14.716	19.924
hsa-miR-4486	13.118	11.386
hsa-miR-4498	11.223	8.735
hsa-miR-4525	4.742	5.249
hsa-miR-4534	7.724	6.604
hsa-miR-4634	5.676	8.037
hsa-miR-4640-5p	12.065	11.072
hsa-miR-4646-5p	20.952	7.933
hsa-miR-4647	3.874	12.679
hsa-miR-4649-5p	3.798	5.867
hsa-miR-4656	32.500	35.659
hsa-miR-4667-5p	11.406	15.501
hsa-miR-4669	5.757	14.703
hsa-miR-4688	4.337	5.385
hsa-miR-4689	6.997	7.704
hsa-miR-4721	9.391	13.980
hsa-miR-4725-3p	5.744	4.751
hsa-miR-4728-5p	12.157	14.302
hsa-miR-4732-5p	12.416	9.641
hsa-miR-4741	5.926	6.804
hsa-miR-4750-5p	16.615	33.833
hsa-miR-4769-5p	2.078	6.008
hsa-miR-483-5p	11.946	12.062
hsa-miR-5006-5p	7.998	15.756
hsa-miR-5010-5p	7.158	5.458
hsa-miR-502-3p	5.443	3.634
hsa-miR-504-3p	8.425	6.446
hsa-miR-5196-5p	12.092	16.465
hsa-miR-5189-3p	0.043	0.037

hsa-miR-5572	11.188	12.291
hsa-miR-5739	13.817	11.224
hsa-miR-574-3p	10.946	11.404
hsa-miR-575	13.865	11.083
hsa-miR-584-5p	5.550	11.325
hsa-miR-6075	7.222	6.004
hsa-miR-6086	6.953	7.093
hsa-miR-6124	12.177	9.363
hsa-miR-6127	16.796	25.564
hsa-miR-6132	11.214	7.042
hsa-miR-6133	5.302	6.867
hsa-miR-6165	13.949	22.103
hsa-miR-625-5p	18.529	18.925
hsa-miR-629-5p	12.958	8.576
hsa-miR-642a-3p	11.686	12.496
hsa-miR-6510-5p	20.297	26.132
hsa-miR-658	6.304	5.705
hsa-miR-664b-5p	17.141	8.377
hsa-miR-665	27.831	40.044
hsa-miR-670-5p	5.556	17.706
hsa-miR-671-5p	11.779	13.182
hsa-miR-6716-5p	29.857	34.464
hsa-miR-6726-5p	9.220	8.068
hsa-miR-6732-5p	0.213	0.305
hsa-miR-6734-5p	9.957	17.352
hsa-miR-6740-5p	4.220	4.324
hsa-miR-6742-5p	4.731	7.398
hsa-miR-6746-5p	6.823	10.714
hsa-miR-6749-5p	4.579	6.526
hsa-miR-6754-5p	19.595	20.118
hsa-miR-6757-5p	10.772	15.799
hsa-miR-6769b-5p	10.676	15.110
hsa-miR-6775-5p	2.337	3.344
hsa-miR-6776-5p	6.267	7.180
hsa-miR-6778-5p	25.056	30.600
hsa-miR-6781-5p	7.156	5.397
hsa-miR-6784-5p	4.911	17.028
hsa-miR-6785-5p	11.271	20.321

hsa-miR-6787-5p	3.326	4.516
hsa-miR-6790-5p	18.872	21.747
hsa-miR-6796-5p	4.360	6.082
hsa-miR-6797-5p	8.627	9.655
hsa-miR-6807-5p	4.385	6.715
hsa-miR-6808-3p	0.138	0.08
hsa-miR-6808-5p	17.725	28.413
hsa-miR-6813-5p	11.565	9.399
hsa-miR-6819-5p	12.729	14.480
hsa-miR-6820-5p	12.664	9.285
hsa-miR-6824-5p	11.096	31.305
hsa-miR-6825-5p	7.229	12.421
hsa-miR-6833-5p	3.226	5.898
hsa-miR-6848-5p	12.352	14.333
hsa-miR-6860	6.032	12.621
hsa-miR-6861-5p	8.240	13.101
hsa-miR-6870-5p	12.196	9.253
hsa-miR-6887-5p	6.364	8.860
hsa-miR-6889-5p	5.412	9.186
hsa-miR-7106-5p	3.808	4.668
hsa-miR-7109-5p	22.634	23.837
hsa-miR-7110-5p	18.472	12.563
hsa-miR-7152-3p	2.325	2.103
hsa-miR-744-5p	43.139	16.035
hsa-miR-7846-3p	20.054	8.223
hsa-miR-7851-3p	15.201	18.398
hsa-miR-8073	4.894	6.343
hsa-miR-8085	3.847	10.349
hsa-miR-885-3p	9.289	12.564
hsa-miR-93-3p	5.382	3.243
hsa-miR-93-5p	5.468	2.999
hsa-miR-939-5p	13.735	14.498
hsa-miR-99b-5p	27.598	20.740

All miRNAs are significantly differentially expressed with both a *p*-value of ≤0.05 and >2-fold-changes.

Supplementary Table S2. List of 12 of exosomal miRNA candidates.

miRNA	Tumor/Normal OSCC patient array (Ratio)	OEC-M1/OKF4-hTERT exosom array (Ratio)	TW2.6/OKF4-hTERT exosom array (Ratio)
hsa-miR-196a-5p	35.63	105.880	44.198
hsa-miR-18a-5p	22.53	183.090	56.841
hsa-miR-421	20.32	14.693	11.292
hsa-miR-1182	16.42	5.179	4.547
hsa-miR-18b-5p	16.06	26.527	6.450
hsa-miR-658	15.37	6.304	5.705
hsa-miR-183-5p	14.81	39.878	19.095
hsa-miR-106b-3p	13.80	29.482	16.579
hsa-miR-32-3p	10.64	4.217	16.420
hsa-miR-885-3p	6.8	9.289	12.564
hsa-miR-130b-3p	5.7	37.953	74.923
hsa-miR-1246	5.6	21.374	10.498

All miRNAs are significantly differentially expressed with both a p-value of ≤ 0.05 and >2-fold-changes.

Supplementary Table S3. List of 27 of miR-421 target genes.

Gene Symbol	PM421 vs. SC	
	Fold-Change	*p*-value
AFF2	-8.61872	0.006645
CPNE2	-2.6044	0.000846
CSNK2A2	-2.20844	0.000026
DTNA	-2.35909	0.045473
GPR176	-2.19421	0.031861
HMGB3	-3.70144	0.000176
HS2ST1	-2.03816	0.006091
HYOU1	-2.27342	0.007428
INPP5A	-2.61028	0.013901
KIAA1549	-2.06549	0.000036
KIF1C	-5.54464	0.005657
KLF11	-2.37498	0.0389
MRAS	-5.27843	0.000026
PMEPA1	-6.07702	0.00058
PREPL	-2.48815	0.021445
PTPN11	-2.24932	0.002898
RAB3B	-3.04331	0.003426
RHOBTB1	-2.1366	0.004817
SCD	-2.89834	0.026678
SERINC5	-2.19874	0.004242
SNX30	-2.32134	0.026373
STX6	-2.0523	0.002471
TMCO3	-2.37306	0.021043
TP53	-2.41363	0.028752
UBASH3B	-2.33547	0.00787
ZCCHC14	-2.29006	0.025959
ZMAT3	-5.5407	0.000028

All genes are significantly differentially expressed with both a *p*-value of ≤0.05 and >2-fold-changes.

Supplementary Table S4. List of primer sequences.

Primer Name	Sequence
Gene Expression Primers	
AFF2-F	CTGACAGCGAATCTAATGAGGC
AFF2-R	CATTGGTTGGATGATTGGAGGA
CPNE2-F	CCTACTGGACCGGGATGTTAC
CPNE2-R	CTCGATCCATCTGCCATTGTT
CSNK2A2-F	GAACCTTCGTGGTGGAACAAA
CSNK2A2-R	CCTGTGCATGATTCCCTTGC
GAPDH-F	GAAGGTGAAGGTCGGAGT
GAPDH-R	GAAGATGGTGATGGGATTTC
GPR176-F	TGGACAGGTACTACTCAGTCCT
GPR176-R	TGGACGTGGCATAGATGTCAG
HS2ST1-F	GCTCCTCAGGATTATGATGCC
HS2ST1-R	TTTCTCGGACTTCGTGTCTTG
HMGB3-F	CCAAAGGGCAAGATGTCCG
HMGB3-R	TTGACAGGGACCTCTGGGTTT
HYOU1-F	GAGGAGGCGAGTCTGTTGG
HYOU1-R	GCACTCCAGGTTTGACAATGG
INPP5A-F	ACTCGGATACCTTAGAGAGCAC
INPP5A-R	TTCTTGACCATTTGCACTCGG
KIAA1549-F	TCAGTGACGAGTAAAGAGGTGG
KIAA1549-R	TGGGCTGACTGTGATATACTGT
KIF1C-F	TCTCGCGTTAGTGAGAACCAG
KIF1C-R	GTTCAAGAGGTCTCGTACCCG
KLF11-F	GCATGACAGCGAAAGGTCTAC
KLF11-R	GGGGTCTTATCCGCAACAGG
MRAS-F	TTCCTCATCGTCTACTCCGTC
MRAS-R	AGGATCATCGGGAATGACTCC
PTPN11-F	GAACTGTGCAGATCCTACCTCT
PTPN11-R	TCTGGCTCTCTCGTACAAGAAA
PMEPA1-F	TGTCAGGCAACGGAATCCC
PMEPA1-R	CAGGTACGGATAGGTGGGC
PREPL-F	AGAAGGTTGTTGCTTGGTTCG
PREPL-R	TCAATGAAGGGCTGGTCTAACT
RAB3B-F	CAACAGCCTATTACCGTGGGG
RAB3B-R	TAGCCCAGTCTTGGACAGCA
RHOBTB1-F	ATGGACGCTGACATGGACTAC
RHOBTB1-R	ATCCCGAGAACGCTCCAAGA
SCD-F	TTCCTACCTGCAAGTTCTACACC
SCD-R	CCGAGCTTTGTAAGAGCGGT
SERINC5-F	CCTGGGTCCAAAATCGACAG
SERINC5-R	GAGAAGGTGAGGTAGGTGACA
SNX30-F	AGCTTCGGTGACAAGGATCTC
SNX30-R	CACACATGCTTCTTGGGATCAT
STX6-F	CACCAACGAGCTGAGAAATAACC
STX6-R	CCCTGACAACTTGCCGAGT

TMCO3-F	TGTGGTGTACTTCTGGGACCT
TMCO3-R	AACACGGCCCTTGTAAGGAAA
TP53-F	CAGCACATGACGGAGGTTGT
TP53-R	TCATCCAAATACTCCACACGC
UBASH3B-F	TCATCGGCCTCTTTGTAAAGGA
UBASH3B-R	ACATGCACTTCGGTTTTGGAT
ZCCHC14-F	GTATTACCCCGTCTTTAAGCAGC
ZCCHC14-R	AGGCACCGTCTCTCTGACT
ZMAT3-F	CCTTACTTCAATCCCCGCTCT
ZMAT3-R	CTTCGCCAGCTCCAACATTAC
DTNA-F	GGCTCACATCGTTGATACTTGG
DTNA-R	GCCACATTCAGGCTGTACTGTA
miRNA Primers	
Universal Reverse	GTGGAGGGTCCGAGGT
RNU-44-RT	GTTGGCTCTGGTGCAGGGTCCGAGGTATTCGCACCAGAGCCA ACAGTCAG
RNU-44-F	GCCCTGGATGATGATAGCAA
miR-421-RT	GTTGGCTCTGGTGCAGGGTCCGAGGTATTCGCACCAGAGCCA ACGCGCCC
miR-421-F	GCGGCGGATCAACAGACATTAATT
Bdi-miR-159-5p-RT	GTTGGCTCTGGTGCAGGGTCCGAGGTATTCGCACCAGAGCCA ACGATTGG
Bdi-miR-159-5p -F	GCGGCGAGCTCCCTTCGAT
LncRNA MEG3 Primers	
MEG3-F	TTGTCTTATTTATTCTCCAACAGCA
MEG3-R	GGCCTTTCAAGAAGCTTGG

Supplementary Figure S1

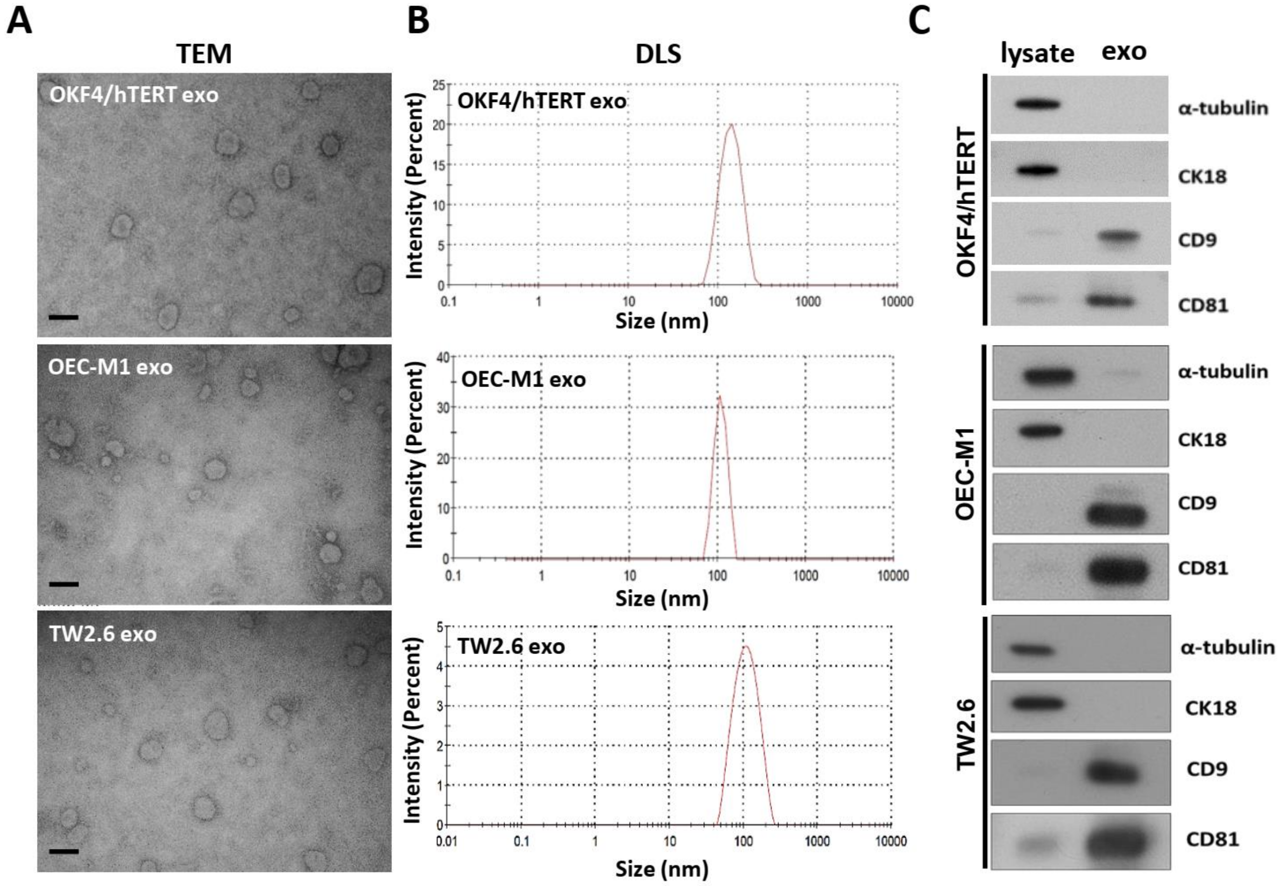

Supplementary Fig. S1 Isolation and characterization of tumor-derived exosomes. (A) Representative images of exosomes isolated from transformed normal human keratinocyte (OKF4/hTERT) and OSCC cells (OCE-M1 and TW2.6) by transmission electron microscope (TEM). Scale bar: 100 nm. (B) The size and quantitation of exosomes examined by dynamic light scattering (DLS). (C) Exosomes (Exo) and total cell lysate were analyzed by Western blotting for cellular proteins (α-tubulin and CK18) and exosome markers (CD9 and CD81).

Supplementary Figure S2

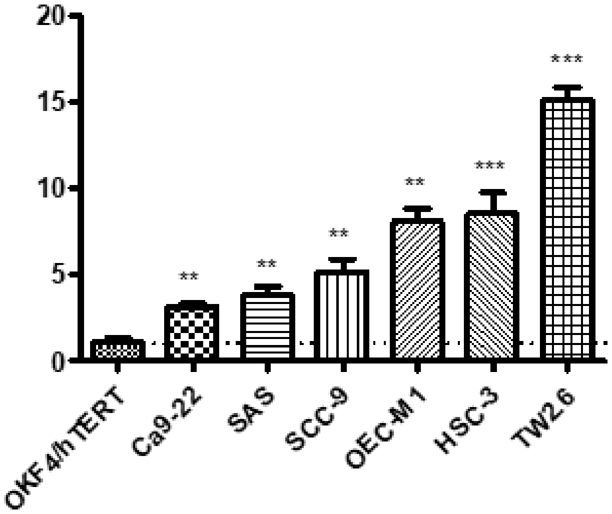

Supplementary Fig. S2 Expression profile of miR-421 in cell lines. MiR-421 expression profile between normal human keratinocyte (OKF4/hTERT) and OSCC cells lines (Ca9-22, SAS, SCC-9, OEC-M1, HSC-3 and TW2.6) by qRT-PCR. Data are represented as mean ± SD and compared with OKF4/hTERT; **$p < 0.01$; ***$p < 0.001$.
